# Overview of Direct and Indirect Effects of Antibiotics on Terrestrial Organisms

**DOI:** 10.3390/antibiotics12091471

**Published:** 2023-09-21

**Authors:** Alessandra Narciso, Anna Barra Caracciolo, Chiara De Carolis

**Affiliations:** 1 Water Research Institute, National Research Council (IRSA-CNR), SP 35d, km 0.7 Montelibretti, 00010 Rome, Italy; alessandra.narciso@irsa.cnr.it (A.N.); chiara.decarolis@irsa.cnr.it (C.D.C.); 2 Department of Ecological and Biological Sciences, Tuscia University, Largo dell’Università s.n.c., 01100 Viterbo, Italy; 3 Department of Environmental Biology, La Sapienza’ University of Rome, P.le Aldo Moro 5, 00185 Rome, Italy

**Keywords:** antibiotic environmental exposure, microbiomes, antibiotic resistance genes, plants, soil fauna, stress response

## Abstract

Antibiotics (ABs) have made it possible to treat bacterial infections, which were in the past untreatable and consequently fatal. Regrettably, their use and abuse among humans and livestock led to antibiotic resistance, which has made them ineffective in many cases. The spread of antibiotic resistance genes (ARGs) and bacteria is not limited to nosocomial environments, but also involves water and soil ecosystems. The environmental presence of ABs and ARGs is a hot topic, and their direct and indirect effects, are still not well known or clarified. A particular concern is the presence of antibiotics in agroecosystems due to the application of agro-zootechnical waste (e.g., manure and biosolids), which can introduce antibiotic residues and ARGs to soils. This review provides an insight of recent findings of AB direct and indirect effects on terrestrial organisms, focusing on plant and invertebrates. Possible changing in viability and organism growth, AB bioaccumulation, and shifts in associated microbiome composition are reported. Oxidative stress responses of plants (such as reactive oxygen species production) to antibiotics are also described.

## 1. Introduction

Antibiotics (ABs) comprise a wide variety of substance classes designed to kill or inhibit microorganisms and their occurrence in ecosystems can significantly alter natural microbial communities [1,2]. Moreover, AB effects on non-target organisms, especially terrestrial ones, are not well known. ABs are used in large quantities to treat or prevent human and animal diseases and in several countries for animal growth promotion [1]. ABs may be natural products, typically bacterial secondary metabolites, semi-synthetic derivatives of natural products, or synthetic substances. Currently 250 different antibiotics are registered for use in human and veterinary medicine [2,3,4]. Once administered, only a part of an AB is metabolized in a treated organism and between 10–100% of this kind of chemical can be excreted in unchanged form or as a metabolite [5]. Consequently, ABs can reach wastewater treatment plants (human origin) and natural environments (livestock origin). Common agricultural practices, such as organic fertilization through manure or biosolid applications, and reclaimed water irrigation [6,7,8], also introduce AB residues to terrestrial ecosystems [9]. Moreover, from soil, antibiotics can reach surface water via runoff and/or be leached to groundwater [10,11]. AB residues in organic waste, such as manure, sewage sludge or biosolids, can range from a few ng/kg to mg/kg soil [6,12,13,14] and agricultural soils are recognized to be an AB reservoir [14,15]. Currently, ABs are considered widespread emerging environmental micro-contaminants because they are present in both water and soil [16,17,18].

AB occurrence in ecosystems can directly alter natural microbial community biodiversity and functioning, with consequences for nutrient cycling and biodegradation of contaminants [16,19,20,21,22,23]. Several studies have underlined the decrease in microbial diversity and inhibition of microbial growth and activities, with consequences for the ecological functions involved in key nutrient transformation [24,25,26,27]. On the other hand, AB residues can modify natural microbial communities, by exerting a selective pressure and promoting the spread of antibiotic resistance gene in ecosystems [28,29,30], with consequences for human and animal health because of antibiotics becoming ineffective [31,32].

Microorganisms are ubiquitous and live in close association with plants and animals, including humans. Animals and plants are no longer viewed as autonomous entities, but rather as “holobionts” [33,34,35], composed of a host plus all of its symbiotic and non-symbiotic microorganisms (microbiomes).

Microbiomes are fundamental to nearly every aspect of host form, function and fitness, including in traits that once seemed untouched by microbiology: behaviour [36,37,38], sociality [39,40], and the origin of species [31]. The conviction that microbiology has a central role in the life sciences has been growing, and microbial symbiosis is becoming a central branch of knowledge in the life sciences [41,42,43]. For this reason, it is crucially important to increase our knowledge of the direct and indirect effects (e.g., on microbiomes) of ABs.

## 2. Environmental Concentrations of Antibiotics in Soils

AB concentrations in the environment vary; their prescribed dosages, treatment times and drug metabolism in a treated organism influence environmental loads. In turn, AB residues in ecosystems depend on their possible degradation. The biodegradation of an antibiotic is linked to the presence of microbial populations resistant to its detrimental effects [44] and at the same time with an acquired ability to degrade it [45,46,47]. Abiotic parameters (such as temperature, water and oxygen availability, and organic matter content) can affect biodegradation [47]. ABs introduced into soils can persist for long periods due to their recalcitrance to degradation or their continuous introduction into the environment (pseudo-persistence), [48]. Indeed, antibiotic residues have different environmental fates in soils depending on their intrinsic characteristics (e.g., solubility and photostability) and they can migrate to water bodies by run-off or leaching or be adsorbed to soil [8,15]. ABs in soils have spatial and temporal variations due to land use, human disturbance, climatic conditions and biological activities [14,49,50,51]. The co-presence of various antibiotics and/or other biocides in soil can influence AB degradation and contribute to the overall toxic effects [3,11,52].

Tetracyclines (TCs), fluoroquinolones (FQs), sulfonamides (SFs), are among the most frequently antibiotics detected in terrestrial ecosystems, because they are widely prescribed in both human and veterinary medicine [53,54,55].

Tetracyclines (TCs), a class of broad-spectrum antibiotics that have as target the bacterial ribosomes [56], have been found at high concentrations in animal manure [12,13,57] and particularly in pig manure [51,58]. Hu et al. [6] detected oxytetracycline (OTC), tetracycline (TET) and chlortetracycline (CTC) in pig manure at concentrations up to 183.5 mg/kg, 43.5 mg/kg and 26.8 mg/kg, respectively. Moreover, a soil fertilized with this manure was analysed and OTC (2.68 mg/kg), TET (0.11 mg/kg) and CTC (1.08 mg/kg) residues were also found, showing how pig manure is an AB source [6,51].

Fluoroquinolones are used to treat various human and animal diseases as they inhibit bacterial DNA synthesis/replication by targeting DNA gyrase and topoisomerase [59]. FQs are known to be persistent compounds [59,60,61]. Ciprofloxacin (CIP), enrofloxacin (ENR) and norfloxacin (NOR) are commonly found in animal excreta. In poultry litter these antibiotics have been detected at concentrations up to 45.6 mg/kg, 1420.8 mg/kg and 225.4 mg/kg for CIP, ENR and NOR, respectively [62]. Other authors found FQs at higher amounts in pig (CIP: 34 mg/kg, ENR: 33.3 mg/kg and NOR: 5.5 mg/kg) than cattle manure (CIP: 29.6 mg/kg; ENR: 46.7 mg/kg and NOR: 2.76 mg/kg [60]. Soil contamination from CIP, ENR and NOR can reach values up to 5.6 mg/kg [14,51,63].

Finally, Sulfonamides act inhibiting nucleic acid synthesis and interfering with folic acid metabolism [51]. Sulfadiazine (SD), and Sulfamethoxazole (SMX), have been also detected in manure, but at lower concentrations (ranging from µg/kg to mg/kg) than FQs [14,51,64]. Consequently, SMX residues in agricultural soil have been found only up to 90 μg/kg [14,51,65,66]. This is because sulfonamides are intrinsically less persistent than FQs. For example, Rauseo et al. [67], in a soil amended with cattle manure, evaluated SMX halving in only 7 days. In this study, the antibiotic sulfamethoxazole, added at an initial high concentration (20 mg/kg) in a soil amended with anaerobically digested cattle manure, displayed an initial detrimental effect on the microbial abundance. At the same time, it promoted a prompt increase in the prevalence of the *intI1* gene (known proxy for environmental antibiotic resistance). A decrease in Sulfamethoxazole over time in a digestate-amended soil was associated with an increase in microbial abundance, suggesting a selection of resistant microbial populations able to biodegrade it.

AB residues in the environment can affect ecosystems in different ways. However the effects on non-target organisms and particularly terrestrial organisms have been poorly investigated so far [68,69,70,71,72]. There is particular interest in the possible effects on the microbiomes of plants and soil fauna. It has been recently recognized that microbiomes associated with plants and other terrestrial organisms contribute to their health status and a biocide can alter these positive interactions [73,74,75].

The present study gives an overview of the direct and indirect effects of ABs, by considering different trophic (e.g., microorganisms, plants and soil fauna) and biological hierarchical levels (from molecular to ecosystem levels), and single species (e.g., plant, worm) and synergic interactions between species (e.g., plant-microbiome system), and ecosystems [76,77,78].

## 3. Antibiotic Effects on Terrestrial Plants

The possibility that antibiotics can cause alterations in plant germination, growth and physiology, and act as an abiotic stress has been recently investigated [79,80,81].

The evaluation of the direct effects of antibiotics on plant germination and growth can be performed applying ecotoxicological studies relying on standard methods (e.g., ISO 11269-2:2012 [82]), using both model target plant species (e.g., *L. sativum*) and common vegetables (e.g., lettuce, rice), and/or soil microcosms [30]. Moreover, the impact of antibiotics can also be evaluated by considering the molecular targets of vegetal cells, in terms of plant genotoxicity [83,84].

For example, Liu et al. [85] evaluated the effects of six antibiotics (chlortetracycline, tetracycline, tylosin, sulfamethoxazole, sulfamethazine) on early growth (germination and root elongation) of different plant species (sweet oat, rice, and cucumber). They evaluated effective concentrations (ECs), in terms of EC_50_, (EC on 50% of the seedlings tested). Chlortetracycline had the highest negative impact on *Oryza sativa* (EC_50_: 16 mg/L) and sulfamethoxazole on *Cucumis sativus* (EC_50_: 8 mg/L). The authors concluded that acute phytotoxic effects varied with the antibiotics and plant species tested.

In another study, Pan et al. [86] assessed whether there was inhibition of root elongation of several edible plants (i.e., lettuce, tomato, cucumber, and carrot) by different antibiotics (tetracycline, sulfamethazine, norfloxacin, erythromycin and chloramphenicol). *Cucumis sativus* was found to be less sensitive to tetracycline (EC_50_: 34.8 mg/L) than chlortetracycline [86]. This result can be ascribed to the presence of the chloride in CTC, which is a known toxic element [87,88]. Among all plants, *Daucus carota* was found to be the most sensitive and tetracyclines to be the most toxic antibiotics for all species tested (Table 1).

More recently, Mukhtar et al. [89] compared five antibiotics, such as levofloxacin, ofloxacin, ciprofloxacin, ampicillin, and amoxicillin separately (at 10 mg/kg each) or combined with different types of organic amendment (i.e., rice husk, poultry litter), to evaluate their acute and chronic effects on *Oryza sativa*. Ciprofloxacin and ofloxacin displayed an acute effect, evaluated in terms of the germination index (IG%). Levofloxacin had only a chronic effect (% Growth inhibition at 4 months), with a decrease in shoot length and biomass. The same authors demonstrated that these effects were alleviated if the organic amendments were present [89].

The overall ecotoxicological results here reported suggest that concentrations effective on plant early growth range from 10 mg/L to values higher than 500 mg/L (Table 1), and these values are much higher than soil residual concentrations, excluding a risk for plant development in a real environmental contamination scenario.

However, the impact of antibiotics has also been evaluated by considering plant genotoxicity, such as DNA damage (e.g., single or double strand breakage). Evans-Roberts et al. [90] hypothesized that the biocide activity of fluoroquinolones, which have bacterial DNA gyrases (an enzyme involved in replication and/or repair in prokaryotes) as targets, can also inhibit chloroplast and mitochondrial enzymes involved in DNA replication. This is because they are similar and have an archetypal prokaryotic enzymatic structure [90,91]. Indeed, Mukhtar et al. [89], performed an ecotoxicological test like the comet assay (OECD 489:2014), and demonstrated that, at 7 days of exposure, fluoroquinolone antibiotics (10 mg/L) caused significant DNA damage to *Oryza sativa* root tips, in comparison with other antibiotics (ampicillin and amoxicillin). On the other hand, the use of organic amendments (e.g., rice husk, poultry litter) reduced antibiotic negative impacts, as mentioned above for the germination endpoint.

Cheong et al. [92] performed a seedling growth test with lentil beans, rice, and *Napa cabbage* and found that sulfamethoxazole, sulfathiazole, sulfadiazine and sulfamethazine had significant effects (at 5 mg/L) on root elongation of the species tested. Because sulfonamides inhibit bacterial dihydropteroate synthase (DHPS), by blocking microbial folate biosynthesis, this result could be ascribed to the effect of these ABs on plant folate metabolism. In fact, these plants have a DHPS similar to the bacterial one.

Considering the detrimental effects of antibiotic residues in soil ecosystems, particular concern also arises about the possibility that plants can uptake, through their roots, antibiotic residues from contaminated soils and bioaccumulate them in their tissue. Consequently, antibiotics can be accidentally ingested by herbivores, entering food chains. This possibility can also involve humans, who consume fresh vegetables [14,93].

Albero et al. [81] simulated exposure conditions in lettuce plant (*Lactuca sativa*) pots by adding organic amendment and spiking a mixture of enrofloxacin, ciprofloxacin, sulfamethazine, sulfamethoxazole and doxycycline, chlortetracycline hydrochloride and lincomycin, (at a concentration of 2.5 mg/kg each). Fluoroquinolones, sulfonamides and lincomycin were detected in plant tissues one month after exposure. Sulfamethoxazole, sulfamethazine and lincomycin were found at the highest concentrations in lettuce shoots with uptake amounts of 0.044, 0.021 and 0.051 mg/kg respectively. These results suggested the various ABs have different bioaccumulation potential in plant tissue, presumably due to their different intrinsic chemical characteristics.

In another study, Cheng et al. [94] evaluated sulfamethoxazole bioaccumulation in lettuce plants, using three different concentrations (100; 200; 300 mg/kg). The antibiotics found in lettuce plants at 120 days of exposure were positively correlated with the initial amount spiked in the soil.

### 3.1. Antibiotics and Oxidative Stress in Plants

The possibility that antibiotics can cause alterations in plant growth and physiology, and act as an abiotic stress has been described in several studies.

The presence of antibiotics can interfere with the photochemical phases of photosynthesis and with electron flows, (both linked to ATP production). A decrease in photosynthesis rates has a consequent impact on plant growth [80]. Negative effects on photosynthesis and an impairment of antioxidant metabolism lead to an increase in the production of reactive oxygen species (ROS) [95]. In response to ROS stress, plants produce enzymes such as catalase (CAT), superoxide dismutase (SOD) and peroxidase (POD) to counteract their presence [80]. In many cases, although there is an increase in production of enzymes, which degrade ROS, this response may be not sufficient to maintain a balance between ROS production and their degradation, and oxidative damage and a consequent growth inhibition occur [80].

Li et al. [79] tested oxytetracycline and enrofloxacin at various concentrations (5, 10, 20, 40 and 80 mg/L) on *Triticum aestivum* L. (wheat), evaluating the plant response in terms of seedling growth, root elongation and antioxidants. No toxic effects were found on seed germination, however, a reduction in root length, fresh weight and surface area was observed, depending on the AB tested (Table 2). From the lowest concentrations of 5 mg/L of ENR and 10 mg/L of OTC, a reduction in plant growth was found. Moreover, the authors also observed that ENR and OTC with concentrations higher than 20 mg/L caused a significant (*p* < 0.05) increase in CAT, SOD, POD activity and malondialdehyde (MDA) content in shoots and roots. The decrease in root and seedling growth was presumably ascribed to a high ROS production. Moreover, at all ENR concentrations tested, an increase in shoots and roots of abscisic acid (ABA), a plant hormone which plays a key role in multiple plant functions, including dealing with environmental stress, was observed. In the case of OTC an increase of ABA content was found only in roots and from 10 mg/L. ABA can be considered an early-warning biomarker. 

Khan et al. [96] analysed the effects on *Brassica rapa* ssp. Chinensis grown in a soil with tetracycline or oxytetracycline or norfloxacin, (100 mg/kg each antibiotic). They found effects on plant growth, chlorophyll, and antioxidant activities. In the TET, OTC, and NOR contaminated soils they found a decrease in plant height of 20%, 12%, and 20%, respectively; fresh weight of 11%, 12%, and 7.0%, respectively and Fv/Fm (Chlorophyll fluorescence parameter) of 9%, 6%, and 2%, respectively, (compared to control plants). Moreover, antibiotic stress increased antioxidant enzyme activities and MDA in shoots of treated plants.

In another study, Jin et al. [97] tested different concentrations (2, 10, 20, 50 mg/L) of ENR, NOR and levofloxacin (LEV) to assess the toxic effects of these quinolones on *Arabidopsis thaliana.* At 7 days, the growth of *A. thaliana* was significantly inhibited, and the leaves turned yellow at 10 mg/L of the antibiotics. The treatment with 50 mg/L ENR resulted in a 33% reduction in fresh leaf weight compared to a control. They observed higher ion leakages of 2.54 (LEV), 2.68 (NOR) and 3.17 (ENR) times than the control at 5.0 mg/L of the antibiotics. Chlorophyll fluorescence parameter (Fv/Fm) values decreased with the increase in AB concentrations. At 2, 10, 20, 50 mg/L of antibiotics, a respective decrease in 5.33%, 56.69%, 57.92%, 73.22% for ENR; 3.42%, 6.42%, 50.96%, 61.48% for NOR; 3.01%, 6.15%, 36.07% for LEV in aged leaves, was observed. In this study, higher relative ROS levels led to an increase in MDA content, with enrofloxacin showing the highest effect. The MDA content in the 50 mg/L ENR treatment was 1.17 times higher than that of levofloxacin.

**Table 1:** Antibiotic effects on plant early growth (germination, biomass, length) and physiology.

Antibiotic	Plant Species	Effect	Reference
Sulfamethoxazole (SMX) Sulfathiazole (STZ) Sulfadiazine (SDZ) Sulfamethazine (SMZ)	*Brassica campestris* *Lens culinaris* *Oryza sativa*	primary root length (EC_50_: 5 mg/L)	[92]
ciprofloxacin (CIP) levofloxacin (LEV) ofloxacin (OFL) amoxicillin (AMX) ampicillin (AMP)	*Oryza sativa*	LEV (10 mg/kg): significant decrease in root/shoot length and biomass reduction CIP (10 mg/kg): significant reduction in seedling vigour index CIP and AMX (10 mg/kg each): significant decrease in root/shoot length and maturity stage. OFL and LEV (10 mg/kg each): reduction in P assimilation All ABs (10 mg/kg each) showed genotoxicity at root tips (DNA damage, evaluated by the comet assay).	[89]
tetracycline (TET) sulfamethazine (SMZ) norfloxacin (NOR) erythromycin (ERY) chloramphenicol (CLP)	1. *Lactuca sativa* 2. *Daucus carota* 3. *Cucumis sativus* 4. *Lycopersicon esculentum*	Root elongation—TET 1. EC_10_: 0.11 mg/L; EC_50_: 14.4 mg/L 2. EC_10_: 0.26 mg/L; EC_50_: 10.3 mg/L 3. EC_10_: 0.43 mg/L; EC_50_: 34.8 mg/L 4. EC_10_: 0.1 mg/L; EC_50_: 11.6 mg/L Root elongation—SMZ 1. EC_10_: 1.94 mg/L; EC_50_: 157 mg/L 2. EC_10_: 25 mg/L; EC_50_ > 300 mg/L 3. EC_10_ > 300 mg/L; EC_50_ > 300 mg/L 4. EC_10_: 5.83 mg/L; EC_50_ > 300 mg/L Root elongation—NOR 1. EC_10_: 0.61 mg/L; EC_50_: 49 mg/L 2. EC_10_: 13 mg/L; EC_50_: 109 mg/L 3. EC_10_: 0.93 mg/L; EC_50_: 75 mg/L 4. EC_10_: 1.1 mg/L; EC_50_: 32 mg/L Root elongation—ERY 1. EC_10_: 0.85 mg/L; EC_50_: 69 mg/L 2. EC_10_: 8.6 mg/L; EC_50_ > 300 mg/L 3. EC_10_: 22.5 mg/L; EC_50_ > 300 mg/L 4. EC_10_: 25.9 mg/L; EC_50_ > 300 mg/L Root elongation—CLP 1. EC_10_: 2.52 mg/L; EC_50_: 204 mg/L 2. EC_10_: 102 mg/L; EC_50_ > 300 mg/L 3. EC_10_: 10.2 mg/L; EC_50_ > 300 mg/L 4. EC_10_: 29 mg/L; EC_50_ > 300 mg/L	[86]
chlortetracycline (CTC) tetracycline (TET) tylosin (TYL) sulfamethoxazole (SMX) sulfamethazine (SMZ)	1. *Oryza sativa* 2. *Cucumis sativus* 3. *Cichaorium endivia*	Root elongation—CTC 1. EC_10_: 0.2 mg/L; EC_50_: 16 mg/L 2. EC_10_: 8 mg/L; EC_50_: 39 mg/L 3. EC_10_: 0.7 mg/L; EC_50_: 48 mg/L Root elongation—TET 1. EC_10_: 14 mg/L; EC_50_: 57 mg/L 2. EC_10_: 16 mg/L; EC_50_: 69 mg/L 3. EC_10_: 8 mg/L; EC_50_: 203 mg/L Root elongation—TYL 1. EC_10_: 19 mg/L; EC_50_: 141 mg/L 2. EC_10_ > 500 mg/L; EC_50_ > 500 mg/L 3. EC_10_: 217 mg/L; EC_50_ > 500 mg/L Root elongation—SMX 1. EC_10_: 16 mg/L; EC_50_: 69 mg/L 2. EC_10_: 0.1 mg/L; EC_50_: 8 mg/L 3. EC_10_ > 300 mg/L; EC_50_ > 300 mg/L Root elongation—SMZ 1. EC_10_: 2 mg/L; EC_50_: 37 mg/L 2. EC_10_: 6 mg/L; EC_50_: 45 mg/L 3. EC_10_: 6 mg/L; EC_50_ > 300 mg/L	[85]
oxytetracycline (OTC) enrofloxacin (ENR)	*Triticum aestivum* L.	OTC (10 mg/L) Reduction in root length (18.6%), biomass (19.8%), surface area (24.8%) and an increase in abscisic acid (ABA) content ENR (5 mg/L) Reduction in root length (29.6%), biomass (32.5%), surface area (35%) and an increase in abscisic acid (ABA) content	[79]
tetracycline (TET) oxytetracycline (OTC) norfloxacin (NOR)	*Brassica rapa* ssp. *chinensis*	TET (100 mg/Kg) 20% plant height reduction; 11% biomass reduction; 9% Fv/Fm reduction OTC (100 mg/Kg) Reduction in plant height (12%); fresh weight (12%) and Fv/Fm (6%) NOR (100 mg/Kg) Reduction in plant height (20%), fresh weight (7%) and chlorophyll fluorescence parameter (Fv/Fm) (2%)	[96]
enrofloxacin (ENR) norfloxacin (NOR) levofloxacin (LEV)	*Arabidopsis thaliana*	ENR Reduction in fresh leaf weight (33%, with 50 mg/L), in chlorophyll fluorescence parameter (Fv/Fm) (5.33%, 56.69%, 57.92%, 73.22% with a concentration of 2, 10, 20, 50 mg/L) and increase in ion leakage (3.17%, with 5.0 mg/L) NOR Increase in ion leakage (2.68%, with 5.0 mg/L) and a reduction in chlorophyll fluorescence parameter (Fv/Fm) (3.42%, 6.42%, 50.96%, 61.48% at a concentration of 2, 10, 20, 50 mg/L) LEV Increase in ion leakage (2.54% with 5.0 mg/L) and a reduction in chlorophyll fluorescence parameter (Fv/Fm) (3.01%, 6.15%, 36.07% at a concentration of 2, 10, 20, 50 mg/L) The MDA content at 50 mg/L ENR was 1.17 times higher than LEV treatment group.	[97]

### 3.2. Antibiotic Effects on Plant-Microbiome System

Soil antibiotic contamination can not only affect plant growth and physiology, but also influence its associated microbiome. Microorganisms are present both on and inside plant tissues, and especially at root level (rhizosphere). The plant microbiome comprises the rhizosphere, phyllosphere (mainly leaves) and endosphere (bacteria which live inside plant tissue). Healthy plants host symbiotic and non-symbiotic rhizo-epiphytic and/or endophytic microorganisms, which do not cause diseases, but support the host nutritionally, by stimulating germination and growth, or helping plants to overcome biotic or abiotic stress [98,99]. Consequently, it is often hard to distinguish any antibiotic effects on a plant from those on its associated microbiome. Moreover, ABs can select antibiotic resistant bacteria and promote the spread of antibiotic resistance genes (ARGs) among soil microorganisms and plant-microbiomes [3,29].

Zhang et al. [100] demonstrated a shift and an increase in ARGs in a lettuce-associated microbiome grown on manure-amended soils. The poultry or cattle manures used in this pot experiment were characterized by a high beta-lactams and tetracycline presence (antibiotics commonly administered in livestock farming). The authors found a change in plant microbiome composition: *Gammaproteobacteria* became dominant in the phyllosphere and *Alphaproteobacteria* in the rhizosphere. Bacteria multi-resistant to these antibiotics were found, particularly at root level (Table 2). Moreover, the same authors found a correlation between some phyla (*Firmicutes*, *Chloroflexi*, *Gemmatimonadetes*, *Acidobacteria*) and the ARGs for aminoglycoside, tetracycline, sulfonamide and beta-lactam resistance. *Proteobacteria* were found to be correlated with a vancomycin resistance gene.

In another study, Wen et al. [101] found a significant correlation between a lettuce microbial community (phyllosphere and endosphere) and tetracycline ARGs. As in the previously cited study, the dominant bacteria class was *Proteobacteria*, and it was correlated with the *tetA* gene. Moreover, the *Chelativorans* genus (*Proteobacteria*) found in the endosphere was correlated to the *intI1* gene (Table 2).

Recently, Yin et al. [102] performed a microcosm experiment with cherry radish simulating an antibiotic treatment for controlling plant pathogens. Streptomycin (STR) or oxytetracycline (OTC), at 1.45 mg/kg each, were added at 21 days of plant growth. The application of the antibiotic was repeated each 6 days for four times. At 44 days of plant growth most of the pots were sampled and eight were maintained with added antibiotic concentrations of 14.5 mg/kg each, in line with agricultural practices for large biomass plants [102]. The ABs did not affect radish growth, and OTC stimulated plant development. However, they found a significant AB accumulation in the radish tissues, mainly in the leaves and fruits at 74 days. Moreover, a shift in the plant microbiome was observed: *Proteobacteria* and *Actinobacteria* increased in leaves and roots. *Cyanobacteria* increased in the rhizosphere.

In a recent study, Barra Caracciolo et al. [30] demonstrated that enrofloxacin and ciprofloxacin bioaccumulated in lettuce leaves grown on manure; moreover, antibiotics resistance genes (*ARGs*) were also found. In particular, the *aac*-(*6*′)-*lb*-*cr* gene related to fluoroquinolone resistance was detected as the most abundant one, followed by *sul1* (for sulfonamides resistance). The same study compared two organic amendments, that is cattle manure and its corresponding digestate. Lettuce plants grown on digestate-amended soil showed a significantly lower AB bioaccumulation and ARG presence than those grown in cattle-manure soil. This study showed how organic amendments can potentially transfer ABs and ARGs to edible plants and then to animals and humans feeding on them. Consuming fresh vegetables which contain AB residues and ARGs can be a risk for organisms and their microbiomes [55].

Finally, an AB presence in a plant-microbiome system can also make plants more sensitive/susceptible to various environmental disturbances [71,103].

Table 2 summarizes data from studies on antibiotic and ARG presence in soil and related plants (soil-plant systems) and/or on the effects in terms of microbial composition shifts.

**Table 2:** Antibiotics and ARGs found in soil-plant systems (1–4 columns) and effects in terms of soil and plant microbiome shifts (5–6 columns).

Antibiotics		ARGs		Microbial Composition		References
Soil	Plant	Soil	Plant Microbiome	Soil	Plant Microbiome	
ABs: 2.5 mg/kg each enrofloxacin (ENR) ciprofloxacin (CIP) sulfamethazine (SMZ) sulfamethoxazole (SMX) doxycycline (DOX) chlortetracycline hydrochloride (CTCC) lincomycin (LIN)	Lettuce: SMX: 0.044 mg/kg fresh weight LIN: 0.051 mg/kg fresh weight SMZ: 0.021 mg/kg fresh weight	-	-	-	-	[81]
Sulfamethoxazole (SMX) 100 mg/kg 200 mg/kg 300 mg/kg	Lettuce: 0.084 mg/kg 0.181 mg/kg 0.503 mg/kg	*sul1* *sul2* *tetM* *tetA/P* *tet34* *tetG1* *tetG2* *qnrS1* *qnrS2* *cmlA1* *floR*	-	Control soil microbial community dominated by: *Gaiella*, *Streptomyces*, *Sphingomonas* Soil + SMX dominated by: *Lysobacter*, *Bacillus*	-	[86]
-	-	beta-lactam, aminoglycoside, MLSB, tetracyclinesulphonamide, FCA, vancomycin, MGEs	beta-lactam, aminoglycoside, MLSB, tetracycline, sulphonamid, FCA, vancomycin, MGEs	Soil: *Actinobacteria* and *Deltaproteobacteria* increase, *Cyanobacteria* decrease in AB presence	Root endophytes: *Alphaproteobacteria* increase and *Gammaproteobacteria* decrease; Phyllosphere: *Actinobacteria* decrease	[100]
Swine manure added to the soil and doxycycline (DOX): 84.02 µg/kg sulfamethoxazole (SMX): 86.41 µg/kg tilmicosin (TIL): 69.37 µg/kg	Lettuce	*tetA*; *tetG*; *tetM*; *tetX*, *intI1*	Phyllosphere: *sul2*; *tetA*; *tetG*; *tetM*; *tetQ*; *ermC* *intI1*; *intI2* Endosphere: *tetQ*; *tetL*; *tetA*; *tetO*; *tetX*; *intI1*; *intI2*	*Fluviicola*, *Cohnella*, *Alcanivorax* bacteria correlated with ARGs	Phyllosphere: *Pseudomonas*, *Clostridium_IV* correlated with ARGs Endosphere: *Chelativorans*, *Halomonas*	[101]
ABs: 7.5 mg/kg each Sulfamethoxazole (SMX) Ciprofloxacin (CIP) Enrofloxacin (ENR)	Lettuce: CIP bioaccumulation, with significantly higher values in manure amended than digestate condition	*sul2* *aac*-(*6*′)-*Ib*-*cr qepA*	Rhizosphere: *sul2* *aac*-(*6*′)-*Ib*-*cr qepA* Phyllosphere: *sul1* *sul2* *aac*-(*6*′)-*Ib*-*cr qepA* *sul1* *tnpA*	Significant increase in *Bacilli* and *Bacteroida* in antibiotic and manure amended conditions Significant decrease in *Actinobacteria* and *Alphaproteobacteria*	Rhizosphere: significant decrease in *Actinobacteria*, *Alphaproteobacteria* and *Bacilli* in antibiotic and manure amended conditions Phyllosphere*:* significant increase in *Bacilli* and *Gammaproteobacteria* in antibiotic and manure amended conditions	[30]
Oxytetracycline (OTC) Streptomycin (STR) Calculated soil concentration of 59.45 mg/kg at 74 days	Cherry radish: Significantly higher presence of STR in plant tissue in comparison with OTC Phyllosphere: STR 0.3 mg/kg OTC 0.003 mg/kg Fruits: STR 0.2 mg/kg OTC 0.005 mg/kg Soil STR 0 mg/kg OTC 0.007 mg/kg	-	-	At 74 days no more *Firmicutes* detected and general decrease in *Actinobacteria.* Increase in *Poteobacteria* and *Chloroflexi* in OTC condition; decrease in *Actinobcteria.* Increase in *Gemmatimonadetes* in STR condition	At 74 days Phyllosphere: decrease in *Bacterioidetes.* Increase in *Cyanobacteria* and decrease in *Firmicutes* in antibiotic conditions. Fruits: increase in *Actinobacteria* in OTC condition. Root Endophytes: increase in *Chloroflexi.* Increase in *Proteobacteria* and *Actinobacteria* in OTC and STR conditions, respectively	[102]

## 4. Effect on Terrestrial Invertebrates

As mentioned above, environmental exposure to antibiotics can influence natural organism growth in different ways. It is still unclear if antibiotics are directly harmful for terrestrial invertebrates, or if they act indirectly by affecting their associated microbiomes [104,105,106]. Antibiotics can display different effects depending on their action mechanisms and on the type of terrestrial organisms exposed [107]. Clarifying which antibiotics are the most detrimental for the most susceptible organisms in an ecosystem has great significance in evaluating the potential ecological hazards of these compounds. Soil fauna (e.g., earthworms), which play a central role in soil nutrient bioavailability by contributing to soil fertility, can be impacted by antibiotic residues from human and organic waste. For example, earthworms can bioaccumulate antibiotics through the ingestion of contaminated fine particles or absorb them through their skin [108].

Zhao et al. [109] exposed earthworms to a multi-antibiotic-contaminated agricultural soil derived from a long-term manure exposure. Twelve antibiotics belonging to the quinolones, tetracyclines and sulfonamide categories were identified, and their concentrations ranged from 0.015 to 0.33 µg/kg for each substance. The results showed a decline in earthworm abundance with increased antibiotic concentrations. Tetracyclines, ofloxacin and sulfamethazine were negatively correlated with earthworm abundance, but only sulfamethazine and oxytetracycline caused a significant decrease in earthworm biomass. A depletion in abundance of earthworms, which can increase soil fertility through their activity and metabolism [110,111,112] can lead to a decrease in nutrient availability for plants, influencing vegetal growth negatively [112].

Parente et al. [113] exposed *Eisenia andrei* earthworms to a soil amended with different quantities of poultry litter contaminated by fluoroquinolones. Ciprofloxacin (CIP) and enrofloxacin (ENR) maximum concentrations in the poultry litter were 6.74 and 23.6 mg/kg, respectively. The authors observed that all individuals tested escaped the contaminated soil, showing so called “avoidance behaviour” [114,115] at 48 h of exposure and for all the concentrations tested. Moreover, they estimated a lethal concentration (LC_50_) at 7 and 14 days and found a significant decrease in biomass. Moreover, dead worms, at the end of the test, showed morphological changes, such as swelling, partition and bottlenecks. The authors also performed chronic tests [116,117], which highlighted a significant effect on worm reproduction (Table 3). Moreover, they found sub-lethal effects on the immune system, in terms of amoebocyte and eleocyte cell variation (e.g., cell density, feasibility and typing). The overall effective concentrations found are reported in Table 3.

Ma et al. [118] studied the bioaccumulation of oxytetracycline, as a model for a commonly used antibiotic for livestock, in the *E. crypticus* earthworm body. The environmental concentration used was 10 mg/kg and animals were exposed to the antibiotic for 21 days. The antibiotic concentration significantly increased in body tissues (45.65 mg/kg) and was significantly higher than in the soil (0.45 mg/kg), showing its bioconcentration. Moreover, the same authors demonstrated a deep change in the *E. crypticus* gut microbiome after antibiotic exposure. *Proteobacteria* relative abundance was affected by oxytetracycline and, in particular, the *Moraxellaceae* family significantly decreased from 15.6% to 2.64%. On the contrary, *Planctomycetes* relative abundance increased, in particular the *Isosphaeraceae* family (from 16.9% to 28.5%). This study showed how the earthworm microbiome can be altered by ABs and can be considered a very sensitive indicator of pollution by them.

Ding et al. [119] chronically exposed the *Drawida gisti* earthworm to a soil amended with either sewage sludge, chicken manure or inorganic fertilizers. The authors analysed the microbiome diversity and ARGs in the earthworm gut. In particular, they evaluated multiple drug resistance, (i.e., the insensitivity or resistance of a microorganism to administered antimicrobials which were structurally unrelated and had different molecular targets) [120]. They found the highest gut microbiome multiple drug resistance, beta-lactam resistance and MLSB (Macrolide-Lincosamide-Streptogramin B) resistance in the chicken manure condition. In the condition with the sewage sludge, the authors reported that beta-lactam resistance increased, while in the chicken manure condition tetracycline resistance increased, in line with the different amendment origin [121]. It is known that TCs because are the most common antibiotics administrated to poultry are found in high amount in poultry manure [122,123]. 

The latter mentioned studies show that not only antibiotic residues, but also ARGs in soil and organic fertilizers may alter the antibiotic resistome in the gut microbiome of soil fauna, making it an additional reservoir of ARGs in terrestrial ecosystems (Table 4) [119].

Zhu et al. [124] performed a more complex experiment using soil fauna, agricultural soils and manure. The authors analysed the changes in the gut microbiome composition of *Collembola*, *Nematodes* and *Enchytraeids*. They found a significant change in all invertebrate microbiomes in response to manure exposure. Notably, ARGs abundance was found to be significantly higher in manure conditions compared to control ones, in all taxa tested.

## 5. Conclusions

Several experimental data show how ABs can have deleterious effects on terrestrial organisms such as plants and soil fauna.

Antibiotics can potentially inhibit seed germination and plant growth; however, the effective concentrations are generally higher than the residual ones found in environment. On the other hand, most common categories of ABs can induce sub-lethal effects at molecular level, such as expression of enzymes linked to stress or DNA damage at concentrations close to environmental ones. The effects found at plant molecular level can be considered biomarkers and early-warning of antibiotic presence in environments. Terrestrial invertebrates have been found more sensitive to ABs than plants. It is still unclear if effects of ABs on soil organisms can be attributed only to the similarity of some target enzymes to bacterial enzymes, or also to the changes of their associated microbiomes. Particular concern relies on the possible presence of AB in crop species because they can have a reduction in productivity and transfer both antibiotics and ARGs to humans and livestock which feed on them. Further studies are needed to clarify these aspects and can be very useful to increase knowledge on ABs and support national and international plans to combat antibiotic resistance. For example, EU National Plans to Combat Antibiotic Resistance have been created with the aim of providing strategic lines and operational indications to face the emergency of antibiotic resistance in the coming years, following a multidisciplinary approach and a One Health vision, promoting constant international data comparison.

## Figures and Tables

**Table 3:** Antibiotic ecotoxicity on terrestrial invertebrates.

Antibiotic	Species	Effect	References
chlortetracycline (CTC) oxytetracycline (OTC) doxycycline (DOX) tetracycline (TET) norfloxacin (NOR) ofloxacin (OFL) lomefloxacin (LOM) ciprofloxacin (CIP) enrofloxacin (ENR) sulfamethoxazole (SMX) sulfamerazine (SMR) sulfamethazine (SMZ)	*Eisenia fetida*	All ABs decreased earthworm abundance (*p* < 0.05) SMZ and OTC also decreased earthworm biomass	[109]
ciprofloxacin (CIP) enrofloxacin (ENR)	*Eisenia Andrei*	CIP LC_50_ (7 days): 0.25 mg/kg LC_50_ (14 days): 0.19 mg/kg biomass decrease (14 days) at 0.27 mg/kg Effect on reproduction at 0.14 mg/kg Immune system cell variation between 0.04 to 14 mg/kg ENR LC_50_ (7 days): 0.89 mg/kg LC_50_ (14 days): 0.67 mg/kg biomass decrease (14 days) at 0.94 mg/kg Effect on reproduction at 0.47 mg/kg Immune system cell variation between 0.12 to 0.47 mg/kg	[113]

**Table 4:** Antibiotic effects on terrestrial invertebrates and their commensal/associated microbiome.

Antibiotics		Organism Microbiome ARGs	Organism Microbiome Microbial Composition	References
Soil Concentration	Organism Concentration			
oxytetracycline (OTC) 10 mg/kg	*Enchytraeus crypticus*: At 21 days 45.65 mg/kg	-	OTC: decrease in *Proteobacteria* relative abundance, *Moraxellaceae* family from 15.6% to 2.64%. *Planctomycetes* relative abundance increase, Isosphaeraceae family from 16.9% to 28.5%.	[118]
tetracycline (TET) oxytetracycline (OTC) chlortetracycline (CTC) doxycycline (DOX) sulfamethoxazole (SMX) sulfadiazine (SDZ) sulfaquinoxaline (SQX) sulfamonomethoxine (SMT) sulfaclozine sodium (SLC) sulfadimethoxine (SMN) sulfameter (SMT) sulfamerazine (SMR) norfloxacin (NOR) ciprofloxacin (CIP) ofloxacin (OFL) enrofloxacin (ENR) roxithromycin (RXM)	*Drawida gisti*	Chicken manure condition: multiple drug resistance, beta-lactam resistance, MLSB (Macrolide-Lincosamide-Streptogramin B) resistance and tetracycline resistance Sewage sludge condition: beta-lactam resistance	-	[119]

## Data Availability

Not applicable.

## Abbreviations

amoxicillin (AMX); ampicillin (AMP); antibiotic resistance genes (ARGs); antibiotics (ABs); chloramphenicol (CLP); chlortetracycline (CTC); chlortetracycline hydrochloride (CTCC); ciprofloxacin (CIP); doxycycline (DOX); enrofloxacin (ENR); erythromycin (ERY); fluoroquinolones (FQs); levofloxacin (LEV); lincomycin (LIN); norfloxacin (NOR); ofloxacin (OFL); oxytetracycline (OTC); streptomycin (STR); sulfadiazine (SD); sulfamethazine (SMZ); sulfamethoxazole (SMX); sulfonamides (SFs); tetracycline (TET); tetracyclines (TCs); tylosin (TYL).
